# Aicardi–Goutières Syndrome associated mutations of RNase H2B impair its interaction with ZMYM3 and the CoREST histone-modifying complex

**DOI:** 10.1371/journal.pone.0213553

**Published:** 2019-03-19

**Authors:** Alexander Shapson-Coe, Brenda Valeiras, Christopher Wall, Cristina Rada

**Affiliations:** MRC Laboratory of Molecular Biology, Francis Crick Avenue, Cambridge, United Kingdom; Texas A&M University, UNITED STATES

## Abstract

DNA-RNA hybrids arise in all cell types, and are removed by multiple enzymes, including the trimeric ribonuclease, RNase H2. Mutations in human RNase H2 result in Aicardi–Goutières syndrome (AGS), an inflammatory brain disorder notable for being a Mendelian mimic of congenital viral infection. Previous studies have shown that several AGS-associated mutations of the RNase H2B subunit do not affect trimer stability or catalytic activity and are clustered on the surface of the complex, leading us to speculate that these mutations might impair important interactions of RNase H2 with so far unidentified proteins. In this study, we show that AGS mutations in this cluster impair the interaction of RNase H2 with several members of the CoREST chromatin-silencing complex that include the histone deacetylase HDAC2 and the demethylase KDM1A, the transcriptional regulators RCOR1 and GTFII-I as well as ZMYM3, an MYM-type zinc finger protein. We also show that the interaction is mediated by the zinc finger protein ZMYM3, suggesting that ZMYM3 acts as a novel type of scaffold protein coordinating interactions between deacetylase, demethylase and RNase H type enzymes, raising the question of whether coordination between histone modifications and the degradation of RNA-DNA hybrids may be required to prevent inflammation in humans.

## Introduction

Aicardi–Goutières syndrome (AGS) is a rare, largely autosomal-recessive disorder characterised by microcephaly, basal ganglia calcification and elevated levels of lymphocytes and interferon-alpha in the cerebrospinal fluid, with occasional extra-neurological involvement of the liver, spleen and skin [1,2]. AGS usually presents at birth or within the first few months of life, and is strikingly reminiscent of congenital viral infection of the brain, although the failure to find a causative pathogen suggests that AGS may be a disorder of the immune system [2]. The AGS phenotype is thought to result from elevated levels of interferon-alpha, and can be recapitulated by overexpression of interferon-alpha in the murine central nervous system leading to the basal ganglia calcification, angiopathy and astrocytosis seen in AGS patients [3].

Consistent with a central role for interferon in the disease, AGS has been associated with heterozygous gain-of-function mutations in the dsRNA sensor Interferon Induced With Helicase C Domain 1 IFIH1 (MDA5), which increase its affinity for RNA as well as baseline and ligand-induced interferon signalling [4]. AGS has also been associated with homozygous or compound mutations in four nucleases; the DNase TREX1 [5], the dsRNA-specific adenosine deaminase ADAR1 [6], the ribonuclease and deoxynucleoside triphosphohydrolase SAMHD1 [7], and all three subunits of the DNA-RNA hybrid-specific ribonuclease RNase H2 [8]. AGS-associated mutations in any one of these enzymes are thought to result in the accumulation of endogenous nucleic acids, which trigger the expression of interferon-alpha and thereby cause the AGS phenotype. However, while it is clear that accumulation of DNA-RNA hybrids can lead to the activation of the innate immune sensing pathways and interferon production [9], the details of how these interferon-stimulatory endogenous nucleic acids accumulate have not yet been fully established. It is particularly intriguing in the case of deficits in RNase H2 associated with AGS, given the existence of several other RNase activities in the cell capable of the removal of RNA/DNA hybrids.

**Fig 1:**
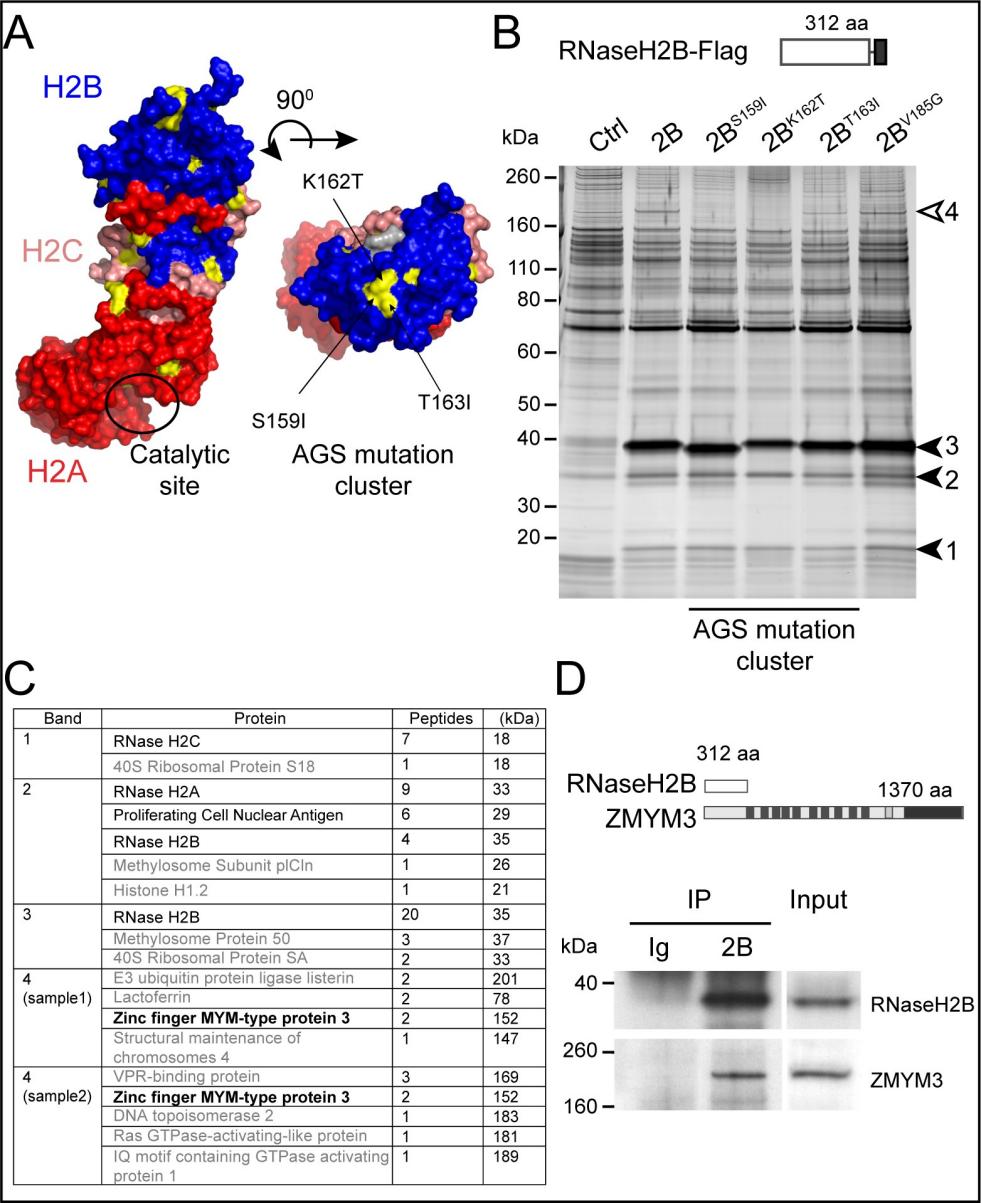
Identification of a novel RNase H2 binding partner ZMYM3. (A) Surface representation of human RNase H2 and location of mutations on the surface of the B subunit. The A, C and B subunits are in red, pink and blue respectively with residues mutated in Aicardi–Goutières syndrome shown in yellow and the catalytic site boxed in black. The location of the S159I, K162T and T163I cluster of mutations are indicated on the top view of the structure. Protein Data Bank accession code: 3PUF [14]. (B) Protein complexes associated with wild-type RNase H2B are disrupted by single amino acid substitutions. Pull down of protein complexes associated with RNase H2B-Flag are shown by silver-stained SDS-Page. Lanes correspond to the individual AGS mutations indicated as well wild type (2B) RNase H2B and nonspecific proteins (Ctrl). The closed arrows indicate the RNase H2B subunits. The open arrow indicates a band that is lost in mutant forms of RNase H2. The same result was observed in three independent pull-downs. (C) Peptides identified by mass spectrometric analysis of the regions corresponding to the arrows indicated in (B), ranked by number of peptides identified. Results for the region corresponding to band 4 from two independent pulldowns are listed separately. The most abundant peptides correspond to the RNase H2 subunits and known associated proteins such as PCNA (in black). The common protein identified in band 4 corresponds to the zinc finger MYM-type protein 3 (bold). (D) Endogenous untagged ZMYM3 is associated with endogenous RNase H2B. The schematic representation indicates the protein size in amino acids (aa). Rabbit anti-RNase H2B immunoprecipitaes from HEK293T cell lysates compared control rabbit IgG from serum. The same result was observed in three independent pull-downs. The input corresponds to ~6% of the lysates used for IP. Rabbit anti-ZMYM3 was developed with goat anti-Rabbit Peroxidase, and the rabbit anti-RNase H2B with TrueBlot anti-rabbit IgG Peroxidase. (Uncropped files are included in S7 Fig).

AGS-associated mutations in RNase H2 may impair its activity on one of two types of DNA-RNA hybrid; DNA-RNA heteroduplexes, including transcription-associated R-Loops [10], and ribonucleotides misincorporated into dsDNA during DNA replication, accumulation of which is associated with the embryonic lethality of RNase H2-null mice [11]. Consistent with this embryonic lethality, no RNase H2-null humans are known to exist, with AGS-associated mutations of the catalytic A subunit reducing but not abolishing catalytic activity *in vitro* [12]. The structure of human RNase H2 shows that the A, B and C subunits are closely intertwined (Fig 1A), and explains why many of the AGS-associated mutations destabilise the complex [13,14]. Consequently, reduced removal of misincorporated ribonucleotides in RNase H2B heterozygous mutants is associated with systemic autoimmunity [15].

However, several AGS-associated mutations of RNase H2 do not affect the catalytic activity of the complex *in vitro* [16,17]. Two of these normal-activity RNase H2B mutants, K162T and V185G, are located on the surface of the complex, and while the V185G mutation is located close to the interface of RNase H2B with the 2A and 2C subunits, the K162T mutation is located within a cluster of AGS-associated mutations comprised of S159I, K162T and T163I, raising the possibility that these residues overlap the binding site of a putative partner of RNase H2 [14]. To further our understanding of the function of RNase H2 that prevents AGS, in the present study we have conducted a screen for novel RNase H2 binding partners that are impaired by catalytically normal AGS-associated mutations, and have identified and characterised a novel interaction between RNase H2 and several members of the CoREST chromatin-silencing complex. This interaction is mediated by the B subunit of RNase H2 and the Mym domain protein ZMYM3 and is impaired by all AGS-associated mutations of the S159I-K162T-T163I cluster of RNase H2B.

## Results

### AGS-associated mutations of RNase H2B impair its interaction with the MYM zinc-finger protein ZMYM3

Previous biochemical and structural studies of RNase H2 suggested the existence of unknown binding partner(s) whose interactions with RNase H2 would be impaired by AGS-associated mutations [14,16,17]. To screen for these putative differential interactors, we transiently expressed FLAG-tagged wild-type and mutant (S159I, K162T, T163I and V185G) (Fig 1A) forms of human RNase H2B in HEK293T cells, immunoprecipitated these along with any interacting proteins, and then compared all binding partners of wild-type and mutant RNase H2 by SDS-PAGE and silver staining, using an empty vector transfection as control. This strategy reproducibly identified a single band, in the wild-type RNase H2B samples that was lost (S159I and K162T) or partially lost (T163I) in the mutants (Fig 1B and S1 Fig). This band identified a single protein of molecular weight between 160kDa and 260kDa which did not match any of the known binding partners of RNase H2.

Bands corresponding to approximately 39kDa, 35kDa and 18kDa (Fig 1B) were excised and as expected were identified by mass spectrometry as RNase H2B, RNase H2A and RNase H2C, respectively (Fig 1C). To focus on identifying the large unknown protein present in the RNase H2B sample but lost in the mutant samples (indicated in Fig 1B by an open arrow), we repeated the pull-down and excised the region between 160kDa and 260kDa from Flag-tagged RNase H2B and controls after denaturing SDS-PAGE and SYPRO RUBY- staining and subjected the excised region to mass spectrometry. Multiple peptides corresponding to the uncharacterised zinc finger protein ZMYM3 were reproducibly recovered from the RNase H2B pull down (Fig 1C) but not in the control sample. The interaction was further recapitulated in HEK293T cells by co-immunoprecipitation of endogenous RNase H2B and ZMYM3 using a previously-validated antibody [18] (Fig 1D). No difference was apparent in the stoichiometry of the A and C subunits co-immunoprecipitated by mutant and wild-type RNase H2B (Fig 1B), confirming previous observations that suggested the AGS associated cluster of mutations in RNase H2B had no significant effect on the stability of the complex [16,17]. To exclude any effect of these mutations on the cellular localisation of RNase H2, wild-type and mutant forms were transiently expressed in HEK293T cells and visualised by confocal microscopy. The nuclei of cells transfected with RNase H2B displayed punctate regions of higher intensity staining (S2 Fig), consistent with previously-described RNase H2-containing replication foci [19], but showed no difference in the localisation of wild-type and mutant RNase H2B (S2 Fig).

**Fig 2:**
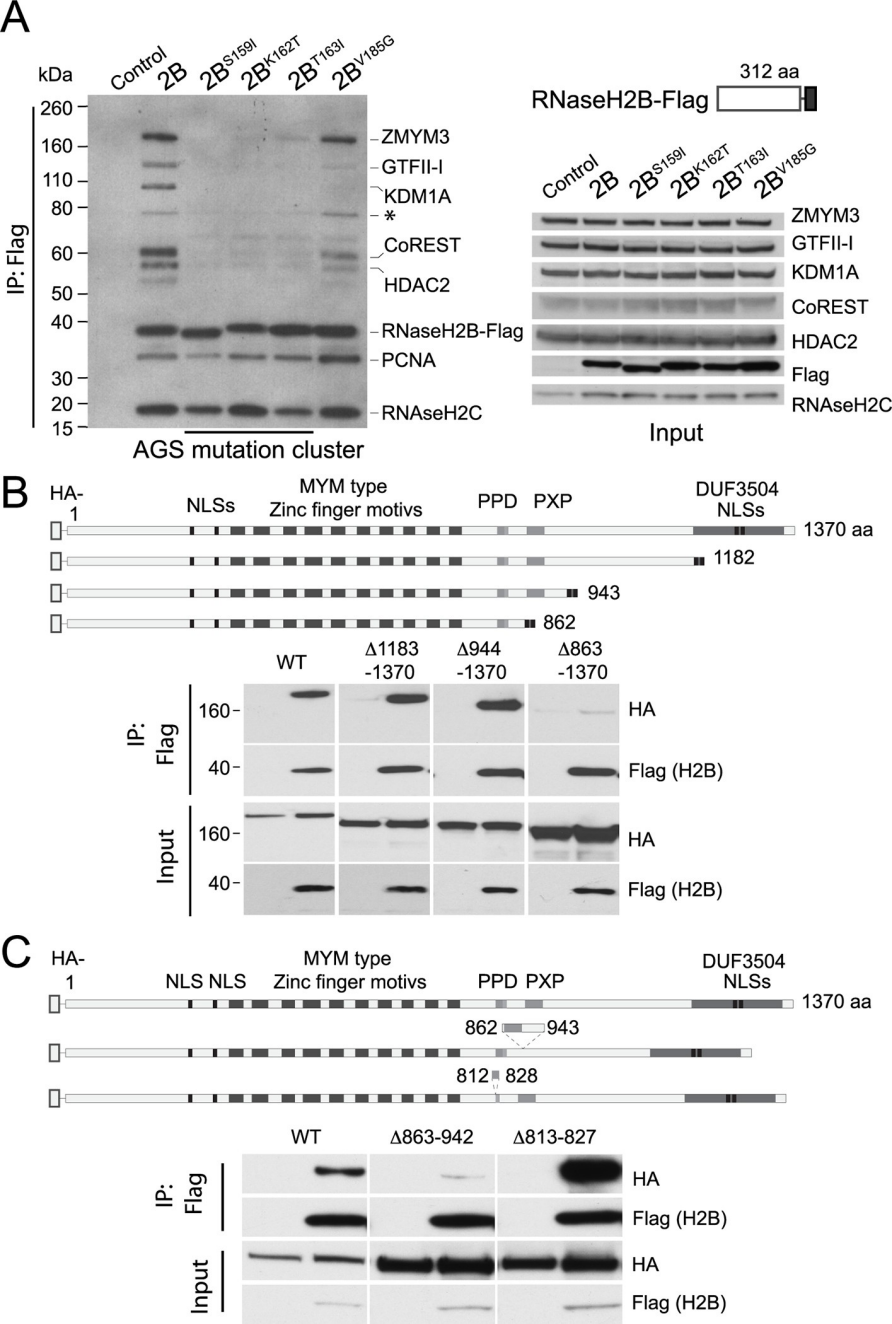
RNase H2 interaction with ZMYM3 and CoREST is mediated the proline rich region. (A) ZMYM3 and RNase H2B are associated as part of the larger CoREST complex. Transfected FLAG-RNase H2B pull downs in HEK293T cell extracts brings down ZMYM3 and CoREST, but both are lost or partially lost in mutants S159I, K162T and T163. On the left, individual developed films are shown as an overlap to allow comparison of the different protein sizes. The input on the right (~3% of the lysates) is shown for the endogenous RNase H and members of the CoREST complex as well as the Flag-RNase H2B transfected protein. Antibodies used for western blotting were Rabbit anti-ZMYM3, Mouse anti-FLAG M2 Peroxidase, Rabbit anti-RNase H2C, Rabbit anti-GFII-I, Rabbit anti-CoREST, Rabbit anti-HDAC2, Goat anti-Rabbit IgG Peroxidase and Goat anti-Mouse IgG Peroxidase. The * indicates an isoform of GTFII-I. The same result was observed in four independent pull-downs. (B) HA-tagged ZMYM3 protein fragments in HEK293T cell extracts co-immunoprecipitated with FLAG-tagged RNase H2B. The schematic shows the domain architecture of ZMYM3 and the C-terminally truncated fragments, including the nuclear localisation region from the DUF3504 domain (black). The first and last amino acids of full and truncation proteins are indicated according to full-length human ZMYM3 (UniProtKB: Q14202.2). PPD: Proline-rich region. NLS: Nuclear localisation signal. Each pull down is shown next to lysates transfected with HA-tagged ZMYM3 fragments and Flag-vector controls. The input equals 3% of the total lysate. Antibodies used to visualise the proteins were Mouse anti-FLAG M2 Peroxidase and Rat anti-HA Peroxidase. (C) Small internal deletions of the conserved proline rich region of motif ZMYM3 prevent its interaction with FLAG-tagged RNase H2B. As B, amino acids shown in the schematic on either side of the deleted segment are according to full-length human ZMYM3 (UniProtKB: Q14202.2). Each pull down is shown next to lysates transfected with HA-tagged ZMYM3 fragments and Flag-vector controls. The input equals 3% of the total lysate. Antibodies used to visualise the proteins were Mouse anti-FLAG M2 Peroxidase and Rat anti-HA Peroxidase. The same results were observed for B and C in three independent pull-downs each.

Having confirmed the interaction between endogenous RNase H2B and endogenous ZMYM3, we next confirmed the loss of interaction in the RNase H2B AGS-associated mutants by western blot (Fig 2A). ZMYM3 is a known interactor of several chromatin-modifying and transcription factors [20], including HDAC2, KDM1A (LDS1), GTFII-I and RCOR1 (CoREST), therefore we also monitored the presence of these proteins in the complex associated with ZMYM3/RNase H2B by western blot. As can be seen in Fig 2A, this revealed each of these proteins to be associated with RNase H2B, with each of these interactions disrupted by each AGS-associated mutation of the S159I-K162T-T163I cluster, and to a lesser degree by the V185G mutation. By contrast, none of the mutations disrupted the well-characterised association of RNase H2B with PCNA [19] (Fig 2A), suggesting that the mutations specifically impair the interaction of RNase H2 with the ZMYM3 protein and the KDM1A/HDAC/CoREST complex.

### RNase H2 binds to a C-terminal region of ZMYM3 containing a ‘PXP’ motif

To establish the region(s) of ZMYM3 responsible for mediating its interaction with RNase H2B, we systematically probed the binding of RNase H2B to C-terminal truncations of ZMYM3 designed on the basis of predicted secondary structure features and domains (Fig 2B). HA-tagged ZMYM3 truncation mutants were co-expressed with FLAG-tagged RNase H2B in HEK293T cells, co-immunoprecipitated with anti-FLAG beads and their binding documented by SDS-PAGE and western blot (Fig 2B and 2C). We identified a region encompassing aa 863 to 942 of ZMYM3 that was required for the co-precipitation of FLAG-RNase H2B and ZMYM3 fragments, and which contained a repeating proline–valine/isoleucine–proline, or PXP motif (see S3 Fig). This was confirmed by internal deletion of aa 863–942 which completely abrogated the interaction, whereas deletion of the neighbouring polyproline domain aa 813–827 had no effect (Fig 2C). Loss of the interaction with the truncation mutants cannot be attributed to a reduction in their abundance (Fig 2C and 2D), nor to a change in their cellular localisation, which was unaffected when transfected in HEK293T and monitored by confocal microscopy (S4 Fig).

### Distinct zinc fingers of ZMYM3 interact with HDAC2, KDM1A, CoREST and GTFII-I

**Fig 3:**
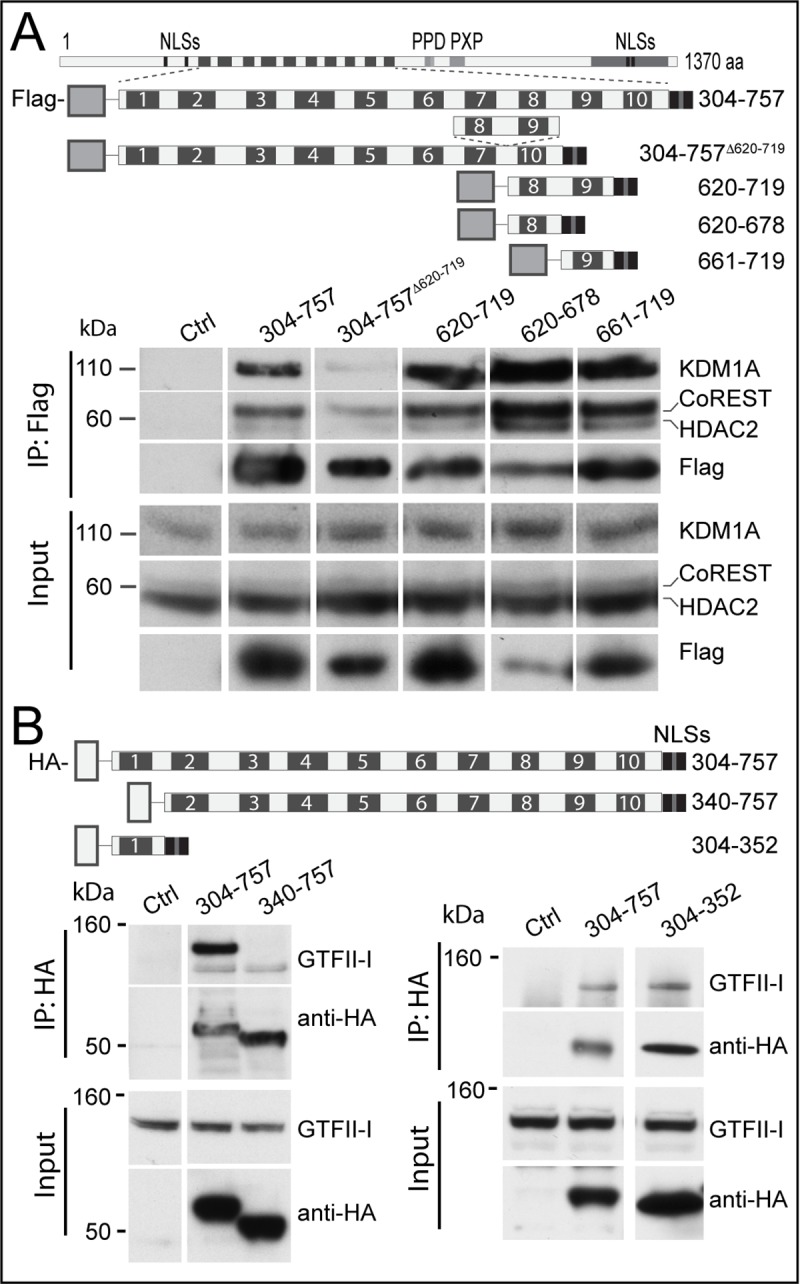
The ZMYM3 zinc finger domains mediate its interactions with the CoREST complex. (A) Pulldown of members of the CoREST complex by FLAG-tagged fragments of ZMYM3 zinc finger domains. The schematic shows the domain architecture of ZMYM3 and the Flag tagged fragments. Lysates from cells transfected with empty vector are show as controls. The input equals 3% of the total lysate. Levels of the endogenous KDM1A, HDAC2 and RCOR1/CoREST proteins relative to each other were consistent in all experiments and are shown as control of each other in input lysates. The expression of fragment 620–678 (corresponding to zinc finger 8) was reproducibly lower than that of the rest of the fragments, including the fragment corresponding to zinc finger 9, as shown. Antibodies used to visualise the proteins were Rabbit anti-CoREST, Rabbit anti-HDAC2, Rabbit anti-KDM1A (LSD1), Goat anti-Rabbit IgG Peroxidase and Mouse anti-FLAG M2 Peroxidase. The size of the molecular standards is indicated in kDa. (B) Pulldown of GFII-I co-immunoprecipitated with HA-tagged fragments of ZMYM3 zinc finger domains. As in A, the schematic shows the fragments identified by the corresponding amino acids. Antibodies used were Rabbit anti-GFII-I, Goat anti-Rabbit IgG-HRP and Rat anti-HA Peroxidase. The same results were observed for A and B in three independent pull-downs each.

The multi-domain nature of ZMYM3 raises the possibility that it could function as a scaffold protein, acting as a platform to bind together RNase H2 and HDAC2, KDM1A, CoREST and GTFII-I. Having established the ZMYM3 binding site of RNase H2, we therefore sought to establish the ZMYM3 binding sites of HDAC2, KDM1A (LSD1), RCOR1 (CoREST) and GTFII-I. As the eighth and ninth zinc fingers of the closely-related ZMYM2 protein have been shown to bind to the complex comprising HDAC1, KDM1A and RCOR1 [21], we sought to establish whether the highly-conserved eighth and ninth zinc fingers of ZMYM3 would play a similar role by constructing a set of N-terminally FLAG-tagged zinc finger arrays (see Fig 3A schematic), all of which were C-terminally appended with the ZMYM3 bipartite NLS contained within the C terminal domain of unknown function (DUF3504) to ensure nuclear localisation. Expression and immunoprecipitation of these zinc finger arrays in HEK293T cells revealed that, as expected, deletion of the eighth and ninth zinc fingers (fragment 304–757^[Δ620–719]^) significantly diminished (but did not abolish) interaction with endogenous RCOR1 and KDM1A, and that either one of these zinc fingers alone (fragments 620–678 or 661–719) was sufficient to bind to RCOR1 and KDM1A (Fig 3A). Using the same assay with HA-tagged zinc finger arrays, we showed that the first zinc finger of ZMYM3 (fragment 304–352) is both necessary and sufficient for the interaction with the transcription factor GTFII-I endogenous protein (Fig 3B).

### RNase H2 can also interact with the ZMYM3 homologues ZMYM2 and ZMYM4

**Fig 4:**
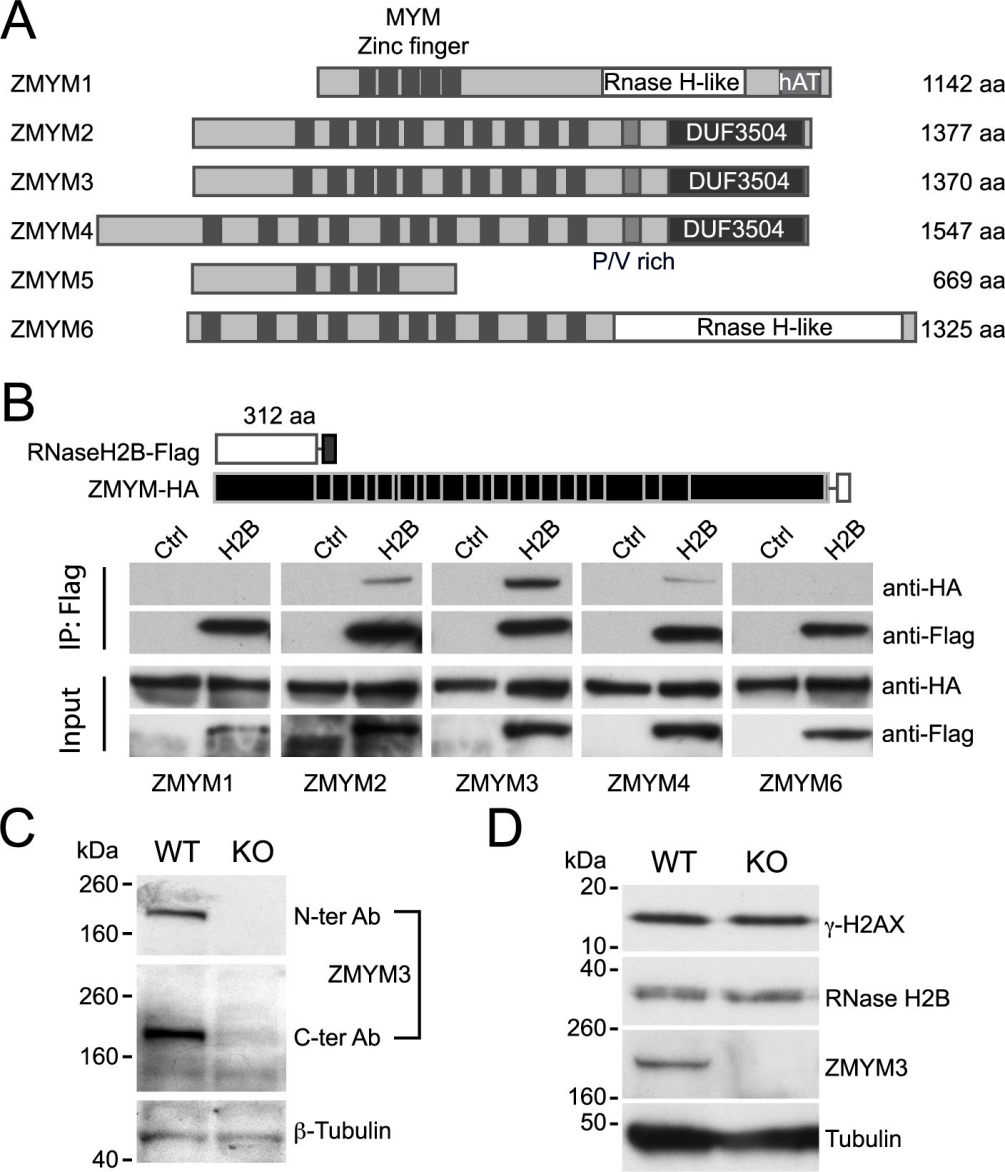
ZMYM3 and RNase H2B interaction is redundant with other ZMYM proteins. (A) Schematic representation of the conserved members of the human ZMYM protein family. Conserved domains are aligned based on homology. The MYM type zinc fingers are indicated as is the proline rich (PV) region and the domain of unknown function DUF3504. ZMYM 1 and 6 contain a domain with structural homology to the RNase H fold, but have no known RNase activity. (B) Pull down of transfected FLAG-tagged RNase H2B and HA-tagged human ZMYM proteins. Empty Flag-vector transfected HEK293T cells lysates are shown as control next to each co-expressing HA- tagged ZMYM 1, 2, 3, 4 and 6 lysates. Antibodies used were Mouse anti-FLAG M2 Peroxidase and Rat anti-HA Peroxidase. The input equals 3% of the total lysate. The same results were observed in three independent pull-downs. (C) Protein expression of mouse ZMYM3 in control and *Zmym3*^*-/*^ ES cells. Two different monoclonal antibodies directed against the N terminus and C terminus of the protein were used to detect ZMYM3 in C57Bl/6 derived ES cells. Targeted *Zmym3*^*-/*^ lacking Exon3 (KO) do not express either portion of the protein. The relative size of molecular standards is shown in kDa. β-tubulin levels are shown as control. (D) Expression of the damage response marker γ-H2AX is not affected in *Zmym3*^*-/*^ ES cells. Protein lysates from the parental C57Bl/6 derived ES cells (WT) compared with the *Zmym3*^*-/*^ ES cell lysates (KO). The relative size of molecular standards is shown in kDa. α-tubulin levels are shown as control. Antibodies used were Goat anti-ZMYM3, Mouse anti-γ-H2AX, Mouse anti-α-Tubulin, Rabbit anti-RNase H2B, Goat anti-Rabbit IgG Peroxidase, Donkey anti-Goat IgG Peroxidase and Goat anti-Mouse IgG Peroxidase. The same results in C and D were observed in three independent clones of targeted C57Bl/6-derived ES cells.

As its name suggests, ZMYM3 is a member of a family of proteins, the six members of which each possess arrays of MYM-type zinc finger motifs [22]. As shown in Fig 2B and 2C, the C-terminal region (aa 862 to 943 including the PXP motif) of ZMYM3 is required for interaction with RNase H2. Presumably ZMYM proteins containing homologous regions may interact with RNase H2, whereas those without should not. As shown schematically in Fig 4A, ZMYM2, ZMYM3 and ZMYM4 have a similar domain architecture and contain homologous proline rich regions in their C-terminal region (see also S3 Fig) whereas ZMYM5 is a shorter protein and ZMYM1 and ZMYM6 contain a different C-terminal region with structural homology to the RNAse-H fold. Using overexpressed HA-tagged proteins we showed that both ZMYM2 and 4 clearly co-immunoprecipitate with FLAG-RNase H2B, whereas ZMYM1 and ZMYM6 do not (Fig 4B). ZMYM5 was very highly expressed and bound non-specifically to beads used for Co-immunoprecipitation of RNase H2B and ZMYM family members, making it impossible to study its interaction (or lack thereof) with RNase H2B. As before, confocal microscopy of transfected ZMYM proteins suggested that the differences in the binding to RNase H2 are not due to lack of subcellular co-localisation, with the possible exception of ZMYM1, which showed a peri-nuclear distribution (S5 Fig).

### ZMYM3 is not required for the stability or genome protection function of RNase H2B in murine ES cells

The results presented above suggested to us that ZMYM3 may act as a scaffold protein to bring together RNase H2 and members of a histone-modifying complex, for the purpose of transcriptional regulation, DNA repair, or other processes. To determine whether ZMYM3 is required for any of these functions, we investigated the *in vitro* phenotype of targeted *Zmym3*^-/^ murine ES cells (see methods and S5 Fig) compared to the parental ES cell line. *Zmym3*^*-/*^ cells cultured under non-differentiation conditions did proliferate, remained viable and retained the ability to differentiate *in vitro* upon retinoid acid induction [23] (S5 Fig). While expression of the ZMYM3 protein was completely lost in the targeted ES cells, we observed no change in the levels of γ-H2AX or RNase H2B, suggesting that the interaction of ZMYM3 with RNase H2 is not required for the role of the latter in maintaining genomic stability in the absence of external damage or cellular stress in ES cells (Fig 4C and 4D).

## Discussion

The experiments presented here identify RNase H2 as a novel component of the co-repressor of transcription KDM1A/RCOR1/HDAC2 LCH complex, via its interaction with ZMYM proteins ZMYM3 and ZMYM2. ZMYM2 and 3 have been shown to associate with the KDM1A/RCOR1/HDAC2 complex [20], although their precise functions within the complex are not known. CoREST was originally identified as a repressor enforcing the transcriptional silencing of neuronal genes in non-neuronal tissues and neuronal stem cells [24] but it also acts on different lineage-specific genes, for example repressing non-erythroid gene expression in haematopoietic stem cells and pro-inflammatory responses in astrocytes [25] or acting to regulate enhancer activity and transcriptional activation of androgen receptor regulated genes in human prostate cell lines [26]. The multiplicity of functions associated with the CoREST complex are a result of the diverse composition of these multiprotein complexes in different cell types and at different developmental stages, but are generally associated with transcriptional silencing and depend on the core KDM1A/RCOR1/HDAC (LCH) complex and its histone H3 lysine4 and lysine9 demethylase activity and histone deacetylase activities [27].

Our results identify several novel interactions of RNase H2, including the uncharacterised zinc finger protein ZMYM3, the transcription factor TFII-I (GTFII-I), the transcription repressor RCOR1, the histone deacetylase HDAC2 and the histone demethylase LSD1 (KDM1A), all of which bind to ZMYM3 [20]. We further identify the domains within ZMYM3 required for the interactions with the components of the CoREST complex. Our data suggest that the zinc finger domain 1 (aa 304–352) mediates the interaction with GTFII-I, quite separately from the zinc finger domains 8 and 9 (aa 620–719) which are required for binding to the main KDM1A/RCOR1/HDAC2/complex, whereas the highly conserved proline rich region in the C terminal domain of ZMYM3, the PXP repeat-containing region (aa 862–949), is sufficient to confer binding to RNase H2.

Although our analyses cannot formally exclude mislocalisation or misfolding of the fragmented proteins used to map the interactions, at least in the case of the interaction with RNase H2B, removal of the PXP repeat-containing region (aa 863–942) either directly (ZMYM3^[Δ863–942]^) or by C-terminal truncation of the protein (ZMYM3^[Δ863–1370]^, ZMYM3^[Δ944–1370]^, ZMYM3^[Δ1124–1370]^, ZMYM3^[Δ1183–1370]^) did not lead to major changes in the nuclear distribution (S4 Fig). In the case of the zinc finger domain1-GTFII-I and the zinc finger domain 8/9-LCH interactions, the interactions are lost by truncation of discrete zinc fingers, which is less likely to result in misfolding due to the ability of zinc fingers of the MYM-type to fold autonomously through their zinc-binding residues [21]. In addition, the fact that isolated zinc finger domain 1 or zinc finger domain 8/9 alone are sufficient to maintain the interaction with GTFII-I and LCH respectively, supports the notion that they are responsible for mediating these interactions in full-length ZMYM3.

While this work clearly implicates the zinc finger domain 8 and 9 of ZMYM3 in binding to KDM1A/RCOR1/HDAC2 LCH complex, the precise architecture of these interactions remains unclear. Previous studies with recombinant ZMYM2, KDM1A/LSD1, CoREST and HDAC1 revealed the interaction of CoREST with ZMYM2 to be dependent on the presence of both KDM1A/LSD1 and HDAC1, and likewise, the interaction of HDAC1 with ZMYM2 to be dependent on the presence of an KDM1A/LSD1-CoREST fusion protein, altogether suggesting that an intact LCH ternary sub complex is required to allow interaction with ZMYM2 [21]. Given the high degree of conservation between ZMYM3 and ZMYM2, and in particular of their LCH-interacting zinc fingers, a similar arrangement seems likely to exist in the case of ZMYM3. However, which of the three LCH members bind directly to the zinc finger domains 8/9 remains to be elucidated for either ZMYM2 or ZMYM3.

The role of the ZMYM3 zinc finger domains 8/9 in binding to KDM1A/RCOR1/HDAC2 could have been anticipated from similar studies of ZMYM2, however, the finding that the zinc finger domain 1 alone is necessary and sufficient for binding to GTFII-I was unexpected but consistent with the structural properties of similar zinc-coordinated domains. As initially described for the zinc-finger LIM domain of zyxin, a single domain is sufficient for its interaction with cysteine-rich protein [28]. Recent data has identified an overlap between the region of ZMYM3 that directly binds dsDNA (aa 300–330) [29] and the region that we identified as binding GTFII-I (aa 304–352), raising the question of whether the DNA binding activity of ZMYM3 is direct. Nonetheless, the fact that GTFII-I and LCH bind to distinct zinc finger domains of ZMYM3 sheds new light on the MYM-type zinc finger domain and the ZMYM3 protein, by showing that it can bring together different proteins in a modular fashion, both within the zinc finger domain region (where individual zinc fingers can act as discrete protein binding modules), and across the protein as whole, with non-zinc finger regions of ZMYM3 (e.g. the PXP-containing region) binding to additional proteins such as RNase H2 (S6 Fig). Although this arrangement suggests a possible scaffolding role for ZMYM3, confirmation of this idea will require further structural studies of the protein and its partners.

By diminishing the interactions of RNase H2B with ZMYM3, it is conceivable that the AGS-mutations investigated here may reduce the stability of the RNase H2 complex, as has been observed for several other AGS-associated RNase H2 mutations that compromise the interaction between the C and A or C and B subunits [13,30]. However, this seems unlikely, as interaction with the A and C subunits is not affected by the mutants in this study, and such a loss of stability would also be expected to affect the abundance of RNase H2B, which is not seen with these mutations (Figs 1 and 2). While the data presented here suggest that loss of interactions between RNase H2 and ZMYM3/GTFII-I/CoREST/HDAC2/KDM1A may result in AGS pathology, and that these interactions may be co-ordinated by ZMYM3, the identification of these interactions raises the more fundamental question: why do they exist at all? This, to our knowledge, is the first time that an RNase H-type enzyme has been shown to interact with a histone-modifying complex, and raises the intriguing possibility that the function(s) of this complex may be dependent on the function(s) of RNase H2, or vice versa. While there is some evidence to suggest that RNase H2 is required for the resolution of R-Loops [10], its best-established role is in the excision of misincorporated ribonucleotides from DNA, failure of which results in catastrophic genomic instability [11,31]. Furthermore, loss of RNase H2 results in accumulation of cytoplasmic aggregates of DNA [32]. The identification of these interactions therefore raises the possibility that the efficient repair of ribonucleotides *in vivo* may require associated changes in chromatin structure (namely, demethylation by KDM1A/LSD1 and deacetylation by HDAC2), or vice versa, and that failure to co-ordinate these processes in humans may contribute to the inflammatory brain disorder AGS. The well-established but functionally poorly understood interaction of lncRNAs or small RNAs with several chromatin remodelling and silencing complexes like CoREST or the NuRD and polycomb complexes [33] might explain the advantages of functional association of such complexes with an RNase H2 enzyme, that can efficiently remove RNA in the context of chromatin.

Failure to coordinate the activity of RNase H2 and the CoREST complex could also lead to an alternative source of nucleic acid accumulation and inflammation, by impairing the ability of the CoREST complex to suppress the expression of endogenous transcripts [25] or by permitting spurious transcription [34,35]. While RNase H2 appears to facilitate, rather than inhibit, LINE-1 retrotransposition [32,36], cell lines with RNase H2 mutations also have significantly reduced overall levels of genomic DNA methylation, compared to cell lines derived from TREX1 or SAMHD1 AGS patients [37], suggesting that silencing of genomic regions (usually correlated with stable DNA methylation) is impaired in cells lacking fully functional RNase H2. Given that mutations of ZMYM3 have been implicated in the pathogenesis of medulloblastoma [38], and intellectual disability syndromes [39,40], it will be interesting to establish whether the loss of this putative scaffolding ability plays a role in either of these pathologies.

Regarding the RNase H2/ZMYM3 interaction, our data suggest that other members of the ZMYM family might act redundantly, since we did not observe mayor abnormalities in mouse ES cells devoid of ZMYM3, unlike the differentiation block associated with KDM1A/LSD1 deficiency [41]. Recent data have shown an alternative function for ZMYM3 in facilitating the repair of DNA damage by homologous recombination by acting as a bridge in the recruitment of BRCA1 to chromatin. Our own data would support the idea of ZMYM3 acting as a bridge to coordinate chromatin remodelling and silencing in the context of ribonucleotide excision repair [29]. It will therefore be important to investigate the association of the RNase H2 with the ZMYM3/CoREST complex in tissues where both CoREST and ZMYM3 are likely to have specific nonredundant functions, such as the brain.

## Methods

### Assembly of RNase H2 and ZMYM constructs

N-terminally FLAG-tagged RNase H2B and mutant versions were assembled by PCR using a cDNA template isolated from a human spleen cDNA library (Life Technologies) and using primers introducing an Nhe I restriction site, KOZAK consensus sequence GCCACC, glycine-alanine linker and FLAG (DYKDDDDK)-encoding sequence 5’ of the protein-coding region (primer 2B N-FLAG Fwd), and a Not I restriction site 3’ of the protein-coding region (primer 2B N-FLAG Rev). Nhe I and Not I PCR products and the vector pIRESpuro3 (Clontech) were digested with NheI and NotI, gel purified, ligated to one another to produce pIRESpuro3-2B (or equivalent mutant). pIRESpuro3-2B (or equivalent mutant) and pCDNA4/TO-IRES-GFP, a mammalian expression vector which expresses the gene of interest and EGFP under the control of a CMV promoter (Addgene), were digested with NdeI and NotI, gel purified (the NdeI/NotI fragment containing the RNase H2B gene and the NdeI/NotI fragment containing the backbone of pCDNA4/TO-IRES-GFP) and ligated to produce pCDNA4/TO-2B-IRES-GFP (or mutant equivalent).

N-terminally HA-tagged ZMYM3 was generated from a cDNA clone of ZMYM3 Isoform 1 (pCMV-SPORT6-ZMYM3; Source Bioscience) as Afl II/Not I fragments with KOZAK/GA linker/HA tag 5’ of the protein-coding region using primers hsZMYM3 N-HA Fwd/hsZMYM3 Rev and expressed in pCDNA4/TO-ZMYM3-IRES-tdTomato. hsZMYM1,2,4 and 6 were isolated from a human spleen cDNA library (Life technologies), as BamH I/Not I PCR fragments containing the KOZAK/GA/HA module using primers hsZMYM1/2/4/6 N-HA Fwd / hsZMYM1/2/4/6 Rev and introduced into pCDNA4/TO-IRES-tdTomato.

ZMYM3 truncation mutants were generated by PCR from pCMV-SPORT6-ZMYM3 as before. All C-terminal truncations (unless otherwise indicated) contained two nuclear localisation signals from the DUF3504 domain of ZMYM3 immediately 3’ of the truncation site while the zinc finger truncation mutants all included the KOZAK/GA/HA module as well as the two predicted nuclear localisation signals from the DUF3504 domain of ZMYM3 and a stop codon. FLAG-tagged ZMYM3 zinc constructs were assembled by PCR as before into pCDNA4/TO-IRES-tdTomato. All primers are listed in S1 Table.

### HEK293T cell culture, transfection and immunoprecipitation

HEK293T cells in Dulbecco's Modified Eagle's Medium (DMEM) supplemented with 100 μg ml^-1^ penicillin and streptomycin (Sigma) and 10% foetal bovine serum (FBS) (Life Technologies) were transfected with 1 μg of plasmid DNA using GeneJuice Transfection Reagent (Novagen) as per the manufacturer’s instructions. Expression of the fluorescent reporter genes was used to monitor transfection efficiency and ~ 4x10^6^ cells were washed in PBS, harvested and lysed in 20 mM HEPES, 150 mM NaCl, 0.2% Triton X-100 (Sigma), 1X Complete Protease Inhibitor (Roche), 1 mM Dithiothreitol, 2 mM MgCl_2_ and 3 μl/ml Benzonase (Novagen) at 4°C for 45 mins under gentle rotation. Cell lysates were precleared by centrifugation 19,000 RCF for 20 mins and 460 μl added to pre-washed 15 μl of anti-FLAG M2 affinity gel (Sigma) with30 μl of each lysate set aside to monitor protein input and incubated at 4°C under gentle agitation. Samples were washed 5x in 500 μl PBS supplemented with 0.1% Triton X-100 (Sigma), centrifuged at 100 RCF for 2 mins in Proteus Clarification Mini Spin Columns (Generon) and the bound proteins eluted 2x in 215 μl 3X FLAG peptide at 10 ng/μl (Sigma) and resuspended in 30 μl NuPAGE LDS Sample Buffer (SB) (Life Technologies) supplemented with 50mM DTT.

For the immunoprecipitation of endogenous RNase H2B and ZMYM3, 10 μl of Dynabeads Protein A beads (Life Technologies) were twice washed in 1ml of RIPA buffer, and resuspended in 100 μl of RIPA, (with 10 μl of 10 μg/μl purified BSA (NEB)) at 4°C for approximately 45mins, washed three times in 1ml of RIPA, and resuspended in 100 μl of RIPA and then added to 460 μl cell lysates in the presence of 1 μg of rabbit anti-RNase H2B or reagent grade IgG from rabbit serum (Sigma) approximately 0°C for two to three hours. Beads were washed 3x in 1ml RIPA buffer (in 1% Triton X-100) and the pellet resuspended in 30 μl NuPAGE LDS Sample Buffer (SB) (Life Technologies) supplemented with 50mM DTT.

### Protein identification

Proteins (typically 20 μl per sample) were separated by SDS-PAGE NuPAGE 4–12% Bis-Tris Gels in 1X NuPAGE MOPS SDS (Life Technologies) and transferred into Immobilin P PVDF membranes (Millipore) in a tris-glycine buffer containing 10% methanol. Membranes were washed 3x5mins in PBS supplemented with 0.05% TWEEN20 (NBS Biologicals) (PBST) under gentle agitation, blocked in PBST supplemented with 5% w/v dried skimmed milk (Marvel) for 45mins and washed 3x5mins in PBST prior to incubation with antibody in PBST supplemented with 2.5% (w/v) milk or PBS at 4°C. Dilutions and antibodies used are specified in S2 Table. Proteins were visualised by ECL Western Blotting Detection Reagents (GE Healthcare), as per the manufacturer’s instructions. Molecular size was estimated from the mobility of Novex Sharp Pre-stained Protein Standards.

For protein identification 35 μl of per sample were separated, gels were fixed and stained using the SilverQuest Staining Kit (Life Technologies), or SYPRO Ruby Protein Gel Stain (Life Technologies), according to the manufacturer’s instructions. Bands of interest were excised and identified by tandem LC-MS/MS mass spectrometry, performed by the mass spectrometry facility at the MRC Laboratory of Molecular Biology, UK.

### Immunofluorescence

Transfected HEK293T cells were grown on poly-l-lysine (Sigma) treated coverslips (Thermo Scientific) for 36 hours and fixed in 1 ml of 4% paraformaldehyde for 20mins at room temperature, washed 3x in 1ml of PBS, permeabilized in 1ml of 0.4% Triton X-100 for 5mins, then washed again in 1ml of PBS 3x 5mins. After blocking in 250 μl of PBS supplemented with 10% normal goat serum (Life Technologies), 0.1% Triton X-100 and 2.5% FBS, cells were stained in 250 μl of blocking buffer for 1hr at room temperature, then washed with 1ml PBS 3x5mins, before being stained with the appropriate dilution (see S2 Table) of secondary antibody in 250 μl blocking buffer for 1hr at room temperature. Coverslips were washed again in 1ml PBS 3x5mins, mounted in 10 μl of Vectashield with DAPI (Vector Laboratories). Confocal microscopy was performed with Zeiss LSM 710 microscope.

### Culture of mouse ZMYM3 targeted embryonic stem cells

Mouse ES cells (C2-C57BL/6NTac) with a targeted inactivation of Zmym3 (*Zmym3*^*tm1(NOCOM)Mfgc*^) were obtained from the NorCOMM consortia (clone N00012P0_C_1W_B6). The targeting of Zmym3 was confirmed by long range PCR and sequencing and Southern Blotting as described previously [42], with 5’ and 3’ probes synthesised from C57BL/6 genomic DNA with the primers 5’ Probe Fwd, 5’ Probe Rev, 3’ Probe Fwd and 3’ Probe Rev (S1 Table). The schematic of the targeting strategy is shown in S5 Fig. Cells were grown on mitomycin-arrested murine embryonic fibroblasts (MEFs) in DMEM (Life technologies) supplemented with 15% FBS (Life technologies), 100 μg ml-1 penicillin and streptomycin (Sigma), 1X LIF (ESGRO) (Millipore), 1X Glutamax (Life technologies), 1% beta-Mercaptoethanol (Life technologies), 1X MEM NEAA (non-essential amino acids) (Life technologies) and 1X Glutamax (Life technologies). Prior to harvesting for protein analyses, cells were grown on gelatine without MEFs in ESGRO-2i media (Millipore), as per manufacturer’s instructions to remove feeder cells and lysates were processed as described for HEK293T cells.

To induce in vitro differentiation, cells were treated according to the 4-/4+ method [23]. Briefly, ES cells were grown on feeders in a 24 well plate, as described until they reached 80% confluence. Cells were washed with 1ml PBS, trypsinized and resuspended in 3 ml of media in the absence of LIF. Cells were grown in No LIF media for four days (‘4-’ phase) at 37°C and 10% CO2, with the media changed daily and for a further 4 days in No LIF media supplemented with 0.5 μM retinoic acid (Sigma) and for a further 8 days in No LIF media without supplementation (day16). The cells were then transferred to serum free N2 media (Life technologies) and cultured for a further 14 days, with the media changed every other day.

## Supporting information

S1 Table Primer sequences. (PDF)

S2 Table Antibodies used. (PDF)

S1 Fig Replicate of co-immunoprecipitation screen for differential interactions of wild-type and AGS-mutated RNase H2B. Protein extracts from control and HEK293T cells transfected with Flag-tagged RNase H2B were incubated with anti-FLAG beads, and the associated proteins were separated by SDS-PAGE and silver stained to reveal any differences in binding between the wild type protein and mutants corresponding to the single amino acid substitutions indicated. An empty vector (control) transfection was also performed to distinguish specific from non-specific binding. The region indicated by the open arrow indicates the region corresponding to the RNase H2B, specific binding identified by mass spectrometry as ZMYM3.
(TIF)

S2 Fig Subcellular localisation of RNase 2B is not affected by the AGS-associated mutations. Confocal micrographs of wild-type and mutant human Flag tagged RNase H2B transfected into HEK293T cells. DAPI stained nuclei and A GFP reporter driven by an IRES was used to monitor transfection and subcellular localisation monitored by staining with M2 anti-Flag antibody and Alexa Fluor 568 Goat anti-Mouse IgG1. A: Wild-type. B: S159I. C: K162T. D: T163I. E: V185G. F: Empty vector control. i: M2 anti-FLAG and. ii: DAPI. iii: GFP expression. iv: Merge. Magnification: 60x.
(TIF)

S3 Fig Conservation of the proline rich region of human ZMYM3. (A) Comparison of the proline rich region in ZMYM3 homologues with identical residues shown in red. The numbering corresponds to the amino acids positions in human (NP_005087), mouse (XP_011245953), zebrafish (XP_005159763) and fly (NP_001097946) proteins. (B) Schematic representation of the domain structure of human ZMYM3 including the secondary structure predictions (pink a-helical regions and green b-sheets) from Phyre2 [43]. Predicted MYM zinc fingers domains (cyan), nuclear localisation signals NLS (black), and the domain of unknown function DUF3504 (blue) are shown. The region responsible for the interaction with RNase H2 is enlarged above, and the degree of conservation between ZMYM2, ZMYM3 and ZMYM4 is indicated by the coloured residues (identical residues in dark red, least conserved in blue). Conservation between ZMYMs 2, 3 and 4 identify a repetitive motif not previously described in ZMYM proteins, the PXP motif (black), comprised of repeats of two prolines interrupted by either an isoleucine or valine residue (i.e. X = I or V). Alignments were performed using online protein sequence aligner PRALINE [44].
(EPS)

S4 Fig Subcellular localisation of ZMYM3 and truncated fragments. The subcellular localization of HA-tagged ZMYM3 and the truncation mutants used to map the biochemical interactions with RNase H2B monitored by confocal microscopy. HEK293T cells stained with Mouse anti-HA antibodies and Alexa Fluor 568 Goat anti-Mouse counterstained with DAPI 24 hours post-transfection. Magnification = 60x. A schematic representation of the full-length protein and the deletion fragments are indicated below the corresponding panels.
(TIF)

S5 Fig Functional redundancy of ZMYM proteins. (A) Confocal micrographs of HA-tagged ZMYM family proteins. HEK293T cells imaged 24 hours post-transfection. Magnification: 60x. Antibodies used include Mouse Anti-HA and Alexa Fluor 568 Goat anti-Mouse. (B) Schematic illustration of the mouse *Zmym3* locus and the NorCOMM targeting strategy. The location of the coding exons is shown as brown boxes and the regions of homology flanking Exon3 used for targeting are shown in blue. Bcl I restriction sites are shown for guidance. The position of the primers used to confirm the correct integration are shown as arrows. The long-range PCR used to monitor the *Zmym*^*3tm(NOCOM)Mfgc*^ allele is shown on the right. (C) Unimpaired *in vitro* differentiation of ZMYM3^-/^ ES cells into neuronal-like cells. In vitro differentiation of *Zmym3*^*-/*^ ES cells following treatment with retinoic acid compared to the C2 parental ES cell line. The time line of the retinoic acid treatment and the time points used for comparison are shown. ES cells were photographed at the times indicated using a Leica DMIL LED Microscope (Leica Microsystems) using a 5x objective, and a QIClick camera and QCapture Suite Plus version 3.1.3.10 (both QImaging).
(TIF)

S6 Fig Schematic representation of ZMYM3 interactions and potential functions of the ZMYM3/RNase H2 interaction. A schematic linear representation of ZMYM3 as a modular scaffold for an array of proteins involved in chromatin modification and recognition. The zinc finger 1 domain is involved in the interaction with General Transcription Factor IIi (GTFII-I) which can recognize DNA in a sequence specific manner (though binding to promoters containing Inr initiator and E-box motifs) whereas the KDM1A/CoREST/HDAC2 LCH complex associates with the central region of the protein through zinc fingers 8 and 9. The C-terminal portion of the protein can recruit RNase H2 to chromatin and DNA though the PXP proline rich domain. This provides a mechanism to coordinate histone tail modification by the LSD1/KDM1A demethylase, histone deacetylation by HDAC2 and transcriptional silencing by CoREST with RNA/DNA hybrid recognition and removal via the RNase activity.
(TIF)

S7 Fig Uncropped source files for main figures. The source images used for each figure are shown and the regions selected to composed the corresponding figures are indicated with the same labelling as used in the main figures.
(PDF)
