# Supplementary material for: Aicardi–Goutières Syndrome associated mutations of RNase H2B impair its interaction with ZMYM3 and the CoREST histone-modifying complex
Source: PLoS One. 2019 Mar 19;14(3):e0213553. doi: 10.1371/journal.pone.0213553 (PMC6424451; doi:10.1371/journal.pone.0213553)
Supplement: S1 Table — (PDF) [file pone.0213553.s001.pdf]

**Table S1. Primer sequences**

| **Name** | **Sequence (5' to 3')** |
|---|---|
| 2B N-FLAG Fwd | TAGCTAGCGCCACCATGGGAGCAGACTACAAGGACGATGACGACAAGGCC GCTGGCGTGGACTGCG |
| 2B N-FLAG Rev | ATGCGGCCGCTCAAACCTTTCCAATTTTTTTTTTATTTTTTACCC |
| S159I Fwd | TATTACAAGTACATCAAAGAGAAGACAT |
| S159I Rev | ATGTCTTCTCTTTGATGTACTTGTAATA |
| K162T Fwd | CAGCAAAGAGACGACATTAAAGTGG |
| K162T Rev | CCACTTTAATGTCGTCTCTTTGCTG |
| T163I Fwd | GCAAAGAGAAGATATTAAAGTGGC |
| T163I Rev | GCCACTTTAATATCTTCTCTTTGC |
| V185G Fwd | ATAATGTGAATGGCAGTTCCCGGGTA |
| V185G Rev | TACCCGGGAACTGCCATTCACATTAT |
| hsZMYM3 N-HA Fwd | CGCTTAAGCCACCATGTACCCATACGATGTTCCAGATTACGCTGGAGCTGA CCCCAGTGATTTCCC |
| hsZMYM3 Rev | CAGCGGCCGCTCAGTCCAGGTCTTCCTC |
| hsZMYM1 N-HA Fwd | CAGGATCCGCCACCATGTACCCATACGATGTTCCAGATTACGCTGGAGCTA AAGAACCACTTTTAGGTGGTGAG |
| hsZMYM1 Rev | CTGCGGCCGCTTATATTTCTTTCATCTGACTGATAAAC |
| hsZMYM2 N-HA Fwd | CAGGATCCGCCACCATGTACCCATACGATGTTCCAGATTACGCTGGAGCTG ACACAAGTTCAGTGGGAGG |
| hsZMYM2 Rev | GTGCGGCCGCTTAGTCTGTGTCTTCATCCAGTTC |
| hsZMYM4 N-HA Fwd | CAGGATCCGCCACCATGTACCCATACGATGTTCCAGATTACGCTGGAGCTG CGGAGAGAGAGGTGGAGTC |
| hsZMYM4 Rev | GTGCGGCCGCTTAATCTGATAATTCAACATCAGAG |
| hsZMYM6 N-HA Fwd | CAGGATCCGCCACCATGTACCCATACGATGTTCCAGATTACGCTGGAGCTA AAGAACCTTTGGATGGTGAATGTG |
| hsZMYM6 Rev | CTGCGGCCGCTACTCTTTCTCCTTCACTAATTTTTC |
| hsZMYM3Δ1124-1370 Rev | CTGCGGCCGCTATTCTCTCTTCCGTTTTCCAGGACCCGTGTCTCGCCCTTTC CTCTGGCGGACTG GAGTGATTTCTCTCACAAACTGGG |
| hsZMYM3Δ944-1370 Rev | CTGCGGCCGCTATTCTCTCTTCCGTTTTCCAGGACCCGTGTCTCGCCCTTTC CTCTGGCGGACTG GCTCAGCCTCTGCAATCATTTCTG |
| hsZMYM3Δ863-1370 Rev | CTGCGGCCGCTATTCTCTCTTCCGTTTTCCAGGACCCGTGTCTCGCCCTTTC CTCTGGCGGACTG GTGGCTTCCACTCTTCTGTTTGAC |
| hsZMYM3Δ1183-1370 Rev | CTGCGGCCGCTATTCTCTCTTCCGTTTTCCAGGACCCGTGTCTCGCCCTTTC CTCTGGCGGACTG GGTTGGGGAGGAGTGTGGGC |
| hsZMYM3ΔPP Rev | CATGGCAGCCTTGTTTTTGCGAGCTGATCGGGTCTTCACAGG |
| hsZMYM3ΔPP Fwd | CCTGTGAAGACCCGATCAGCTCGCAAAAACAAGGCTGCCATG |
| hsZMYM3Δ863-942 Rev | CAGATGAGGCCTTGTCTAACTCTGGCTTCCACTCTTCTGTTTGAC |
| hsZMYM3Δ863-942 Fwd | GTCAAACAGAAGAGTGGAAGCCAGAGTTAGACAAGGCCTCATCTG |
| hsZMYM3-HA-ZF1 Fwd | GCGAATTCGCCACCATGTACCCATACGATGTTCCAGATTACGCTGCAGTGG GCACCAAGATGAC |
| hsZMYM3-HA-ZF2 Fwd | GCGAATTCGCCACCATGTACCCATACGATGTTCCAGATTACGCTCTCACCAC TTTCTCCAAGAAG |
| hsZMYM3-NLS-ZF1 Rev | GCGGCCGCTATACCTTACGTTTCTTCTTAGGGGTCTTTTTGCCCGAGGGC |
| hsZMYM3-NLS-ZF8 Rev | GCGGCCGCTATACCTTACGTTTCTTCTTAGGAGTGATACAGCACAAGCCCA G |
| hsZMYM3-NLS-ZF9 Rev | GCGGCCGCTATACCTTACGTTTCTTCTTAGGCCGGGCAGCCTTGCAGTAC |
| hsZMYM3-NLS-ZF10Rev | GCGGCCGCTATACCTTACGTTTCTTCTTAGGTTGGTTCTGCTGGCTATAGAA AC |

| | |
|---|---|
| hsZMYM3-FLAG-ZF1 Fwd | GACTTAAGCCACCATGGACTACAAGGACGATGACGACAAGGCAGTGGGCACCAAGATGAC |
| hsZMYM3-FLAG-ZF8 Fwd | GACTTAAGCCACCATGGACTACAAGGACGATGACGACAAGTGTGAGGACTTCAAGCGGCTTC |
| hsZMYM3-FLAG-ZF9 Fwd | GACTTAAGCCACCATGGACTACAAGGACGATGACGACAAGGTGCTGCTGTACAAACAGGAC |
| ΔZF89 Fwd | CTTCGGGGTGTGGTGTCCCAGAAGAGCAAGTACCTGCTGTGG |
| ΔZF89 Rev | CCACAGCAGGTACTTGCTCTTCTGGGACACCACACCCCGAAG |
| 5’ HA Fwd | GCGATCGCGTTGCTGTCTGCAGTTCTAGG |
| 5’ HA Rev | TGTACAGAATGGGAAAACTTGAGTGCCTG |
| 3’ HA Fwd | GCGGCCGCTCCCGTGTTTACTGATTCTTAG |
| 3’ HA Rev | GTTTAAACAGACCCCATGTCAAGAAGGC |
| Ex3 Fwd | TGTACATGATCAGTTTGGAGTAAATTGTTC |
| Ex3 Rev | GCGGCCGCATAACTTCGTATAATGTATGCTATACGAAGTTATTCTGACAGCACCCCCCCAAC |
| 5’ Probe Fwd | CTCCTACAGGAGGATTCAGTG |
| 5’ Probe Rev | GAGGAGGGGGCTCGCGAG |
| 3’ Probe Fwd | TGGCCCAGAAAGTCTTGTGTC |
| 3’ Probe Rev | CAGGATCAGCTGATGAGAGAG |
| ZESC2 | AGCTGACACTAGTGGCAGGC |
| ZESC3 | CTTGCTCCTTTTAAGTGGTTAC |
| ZESC4 | TGCCAGTTTTCCAGATTAAGGC |
| ZESC5 | TTCCCCACAACGGGTTCTTC |
