# Supplementary material for: Aicardi–Goutières Syndrome associated mutations of RNase H2B impair its interaction with ZMYM3 and the CoREST histone-modifying complex
Source: PLoS One. 2019 Mar 19;14(3):e0213553. doi: 10.1371/journal.pone.0213553 (PMC6424451; doi:10.1371/journal.pone.0213553)
Supplement: S2 Table — (PDF) [file pone.0213553.s002.pdf]

Table S2. Antibodies

Name	Host	Clonality	Conditions	Source
Rabbit anti-ZMYM3	Rabbit	Polyclonal	Western Blotting: 1:5000	Bethyl (A300-200A)
Rabbit anti-RNase H2B	Rabbit	Polyclonal	Western Blotting: 1:1000	Abcam (ab122619)
Rabbit anti-RNase H2C	Rabbit	Polyclonal	Western Blotting: 1:1000	Abcam (ab89726)
Rabbit anti-GTFII-I	Rabbit	Polyclonal	Western Blotting: 1:50	Abgent (AP6881b)
Rabbit anti-LSD1	Rabbit	Polyclonal	Western Blotting: 1:100	Abgent (AP1218c)
Rabbit anti-HDAC2	Rabbit	Polyclonal	Western Blotting: 1:500	GeneTex (GTX112957)
Rabbit anti-CoREST	Rabbit	Polyclonal	Western Blotting: 1:2000	Millipore (07-455)
M2 anti-FLAG	Mouse	Monoclonal	Immunoflourescence: 1:1000	Sigma (F3165)
Alexa Fluor® 568 Goat anti-Mouse	Goat	Monoclonal	Immuno-flourescence: 1:400	Life Technologies (A21124)
Mouse anti-FLAG M2 Peroxidase	Mouse	Monoclonal	Western Blotting: 1:15000	Sigma (A8592)
Goat anti-Rabbit IgG Peroxidase	Goat	Monoclonal	Western Blotting: 1:30000	Novus Biologicals (NB730-H)
TrueBlot anti-Rabbit IgG Peroxidase	Goat	Monoclonal	Western Blotting 1:1000	eBioscience (18-8816)
Goat anti-Mouse IgG Peroxidase	Goat	Monoclonal	Western blotting: 1:10000	Novus Biologicals (NB7539)
Donkey anti-Goat IgG Peroxidase	Donkey	Monoclonal	Western Blotting: 1:10000	Novus Biologicals (NB7539)
Mouse anti-HA	Mouse	Monoclonal	Immuno-flourescence 1:200	Sigma (H9658)
Rat anti-HA Peroxidase	Rat	Monoclonal	Western Blotting: 1:1000	Roche (12013819001)
Mouse anti-γ-H2AX	Mouse	Monoclonal	Western Blotting: 1:1000	Millipore (05-636)
Mouse anti-α-Tubulin	Mouse	Monoclonal	Western Blotting: 1:1000	Abcam (ab7291)
