# Supplementary figures and images for: Aicardi–Goutières Syndrome associated mutations of RNase H2B impair its interaction with ZMYM3 and the CoREST histone-modifying complex
Source: PLoS One. 2019 Mar 19;14(3):e0213553. doi: 10.1371/journal.pone.0213553 (PMC6424451; doi:10.1371/journal.pone.0213553)

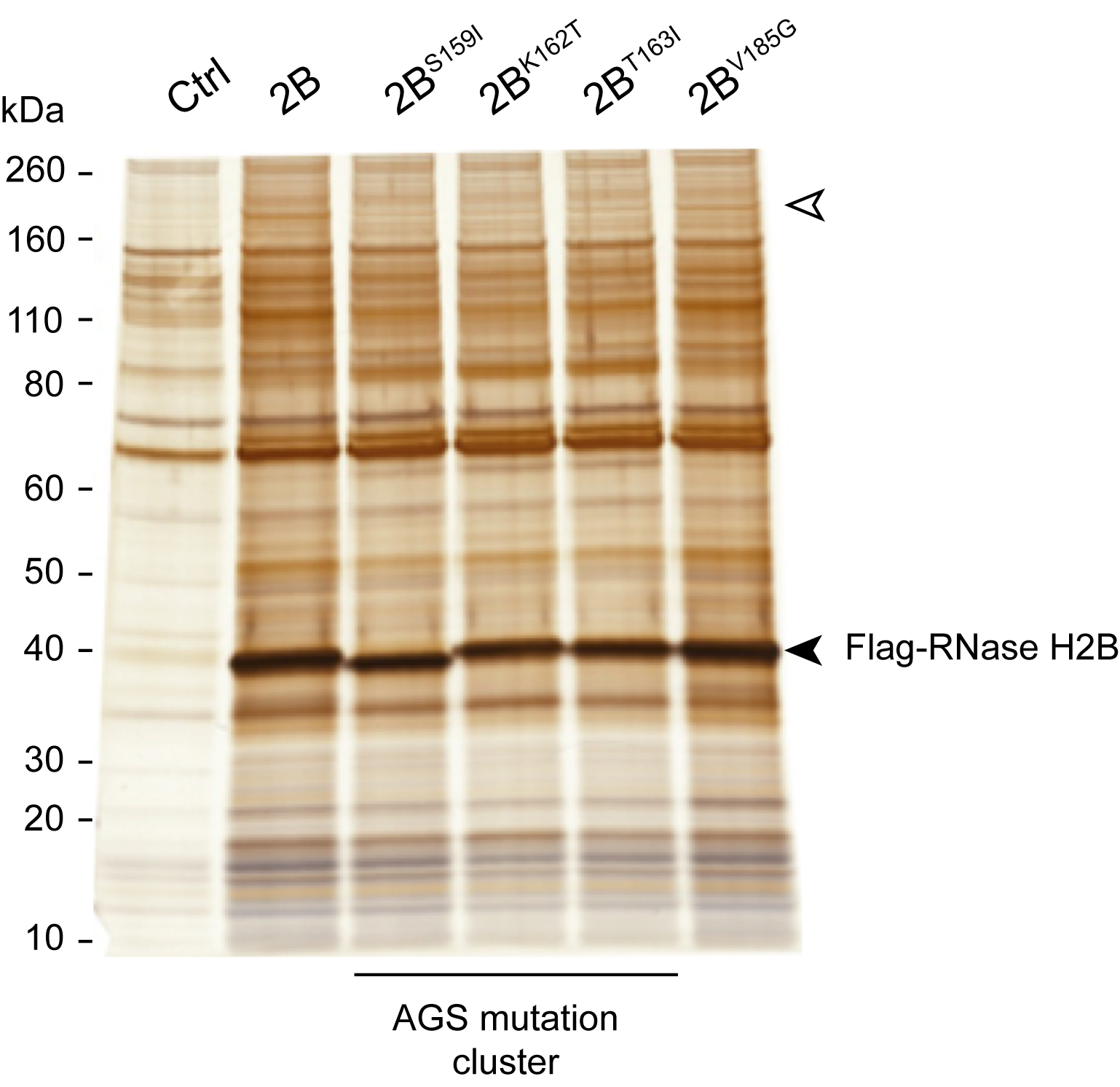

Supplement: S1 Fig — Protein extracts from control and HEK293T cells transfected with Flag-tagged RNase H2B were incubated with anti-FLAG beads, and the associated proteins were separated by SDS-PAGE and silver stained to reveal any differences in binding between the wild type protein and mutants corresponding to the single amino acid substitutions indicated. An empty vector (control) transfection was also performed to distinguish specific from non-specific binding. The region indicated by the open arrow indicates the region corresponding to the RNase H2B, specific binding identified by mass spectrometry as ZMYM3. (TIF) [file pone.0213553.s003.tif]

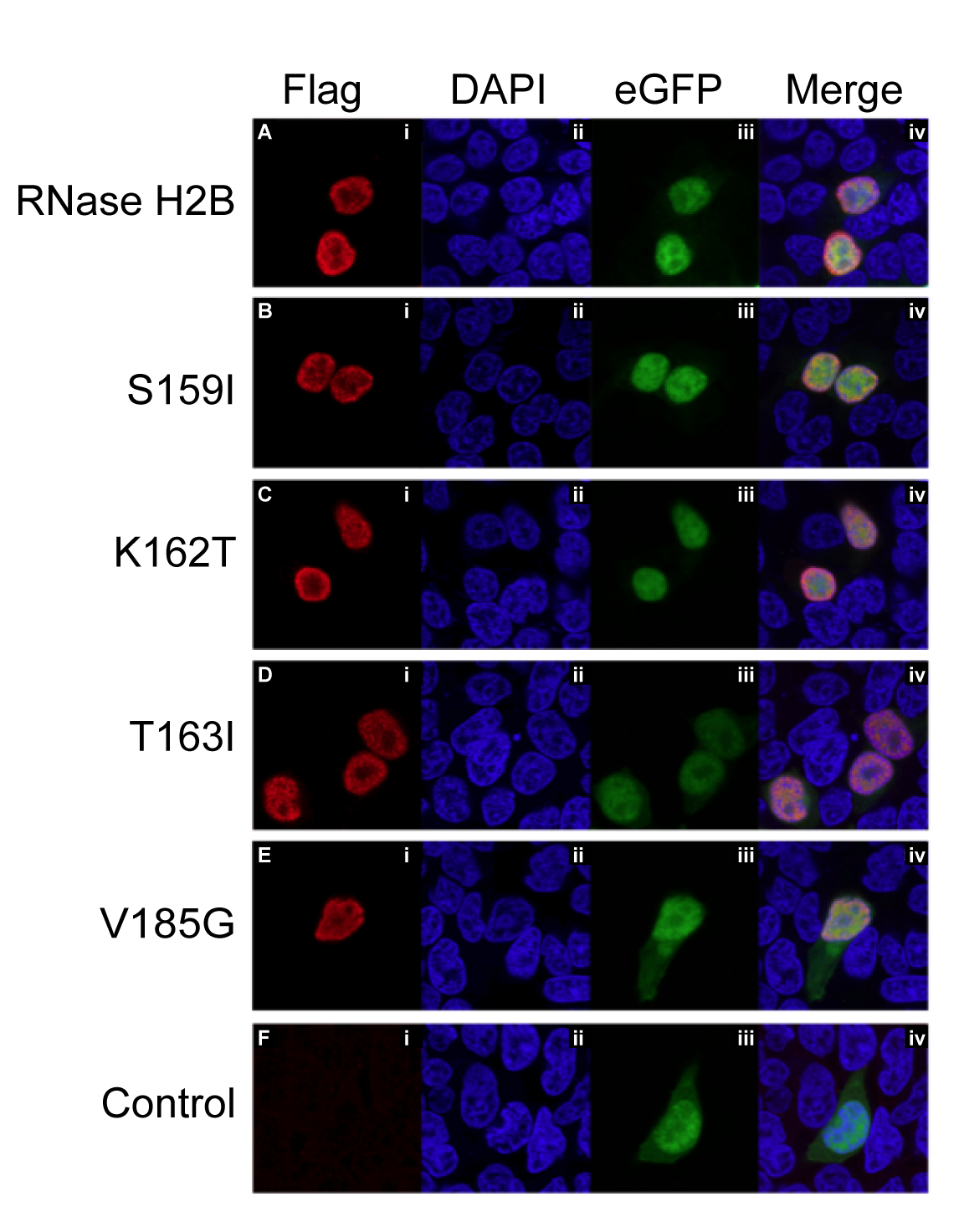

Supplement: S2 Fig — Confocal micrographs of wild-type and mutant human Flag tagged RNase H2B transfected into HEK293T cells. DAPI stained nuclei and A GFP reporter driven by an IRES was used to monitor transfection and subcellular localisation monitored by staining with M2 anti-Flag antibody and Alexa Fluor 568 Goat anti-Mouse IgG1. A: Wild-type. B: S159I. C: K162T. D: T163I. E: V185G. F: Empty vector control. i: M2 anti-FLAG and. ii: DAPI. iii: GFP expression. iv: Merge. Magnification: 60x. (TIF) [file pone.0213553.s004.tif]

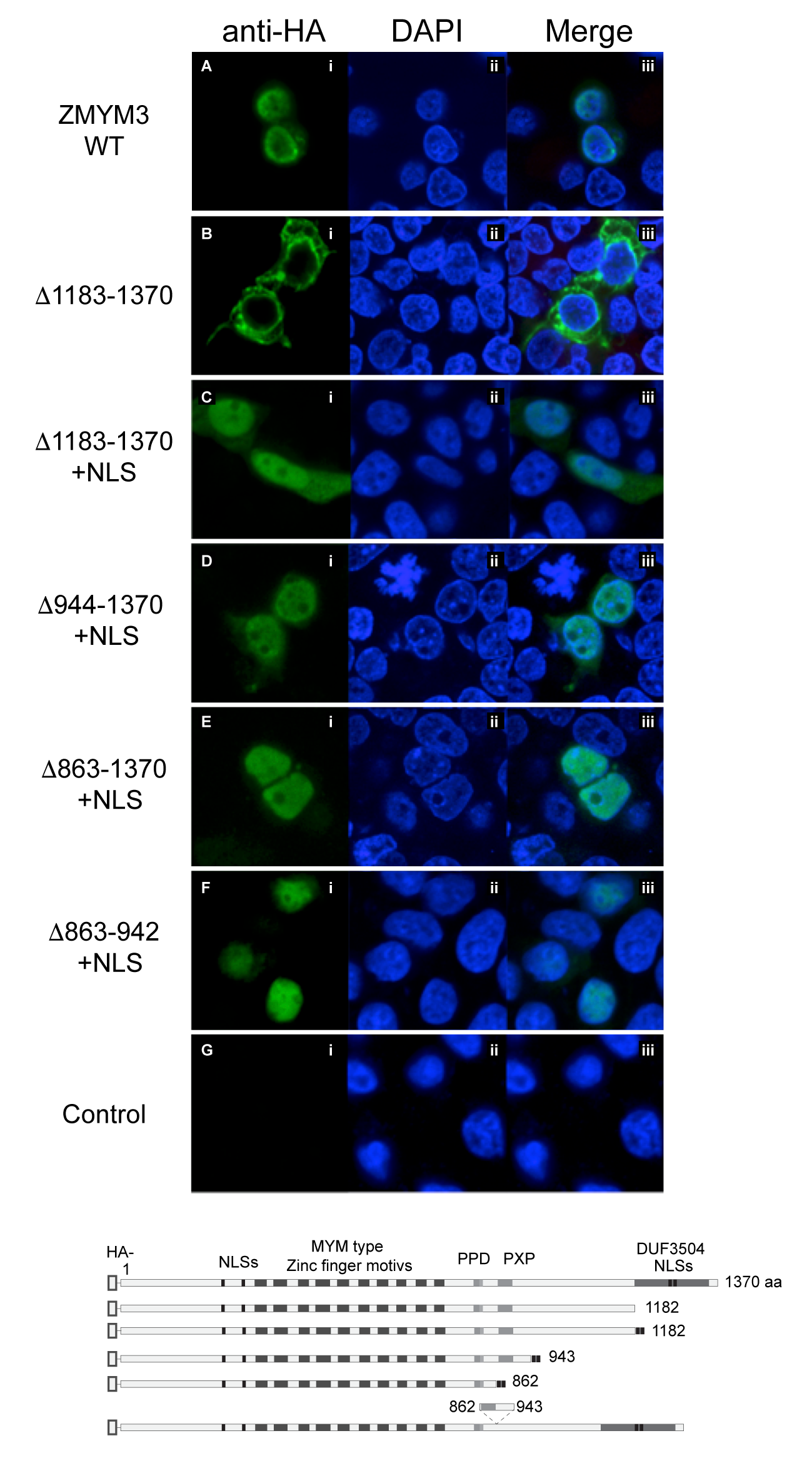

Supplement: S4 Fig — The subcellular localization of HA-tagged ZMYM3 and the truncation mutants used to map the biochemical interactions with RNase H2B monitored by confocal microscopy. HEK293T cells stained with Mouse anti-HA antibodies and Alexa Fluor 568 Goat anti-Mouse counterstained with DAPI 24 hours post-transfection. Magnification = 60x. A schematic representation of the full-length protein and the deletion fragments are indicated below the corresponding panels. (TIF) [file pone.0213553.s006.tif]

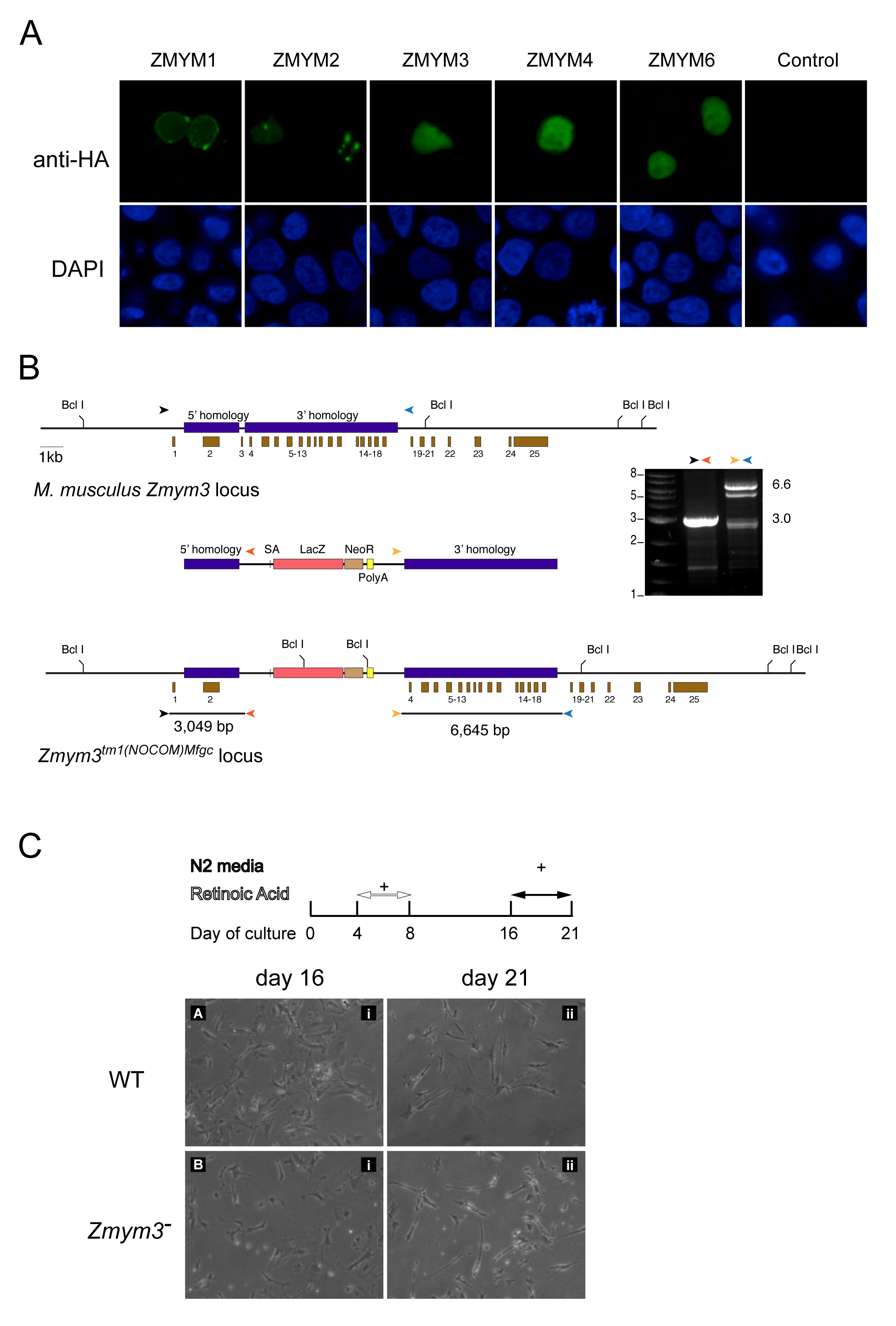

Supplement: S5 Fig — (A) Confocal micrographs of HA-tagged ZMYM family proteins. HEK293T cells imaged 24 hours post-transfection. Magnification: 60x. Antibodies used include Mouse Anti-HA and Alexa Fluor 568 Goat anti-Mouse. (B) Schematic illustration of the mouse Zmym3 locus and the NorCOMM targeting strategy. The location of the coding exons is shown as brown boxes and the regions of homology flanking Exon3 used for targeting are shown in blue. Bcl I restriction sites are shown for guidance. The position of the primers used to confirm the correct integration are shown as arrows. The long-range PCR used to monitor the Zmym3tm(NOCOM)Mfgc allele is shown on the right. (C) Unimpaired in vitro differentiation of ZMYM3-/ ES cells into neuronal-like cells. In vitro differentiation of Zmym3-/ ES cells following treatment with retinoic acid compared to the C2 parental ES cell line. The time line of the retinoic acid treatment and the time points used for comparison are shown. ES cells were photographed at the times indicated using a Leica DMIL LED Microscope (Leica Microsystems) using a 5x objective, and a QIClick camera and QCapture Suite Plus version 3.1.3.10 (both QImaging). (TIF) [file pone.0213553.s007.tif]

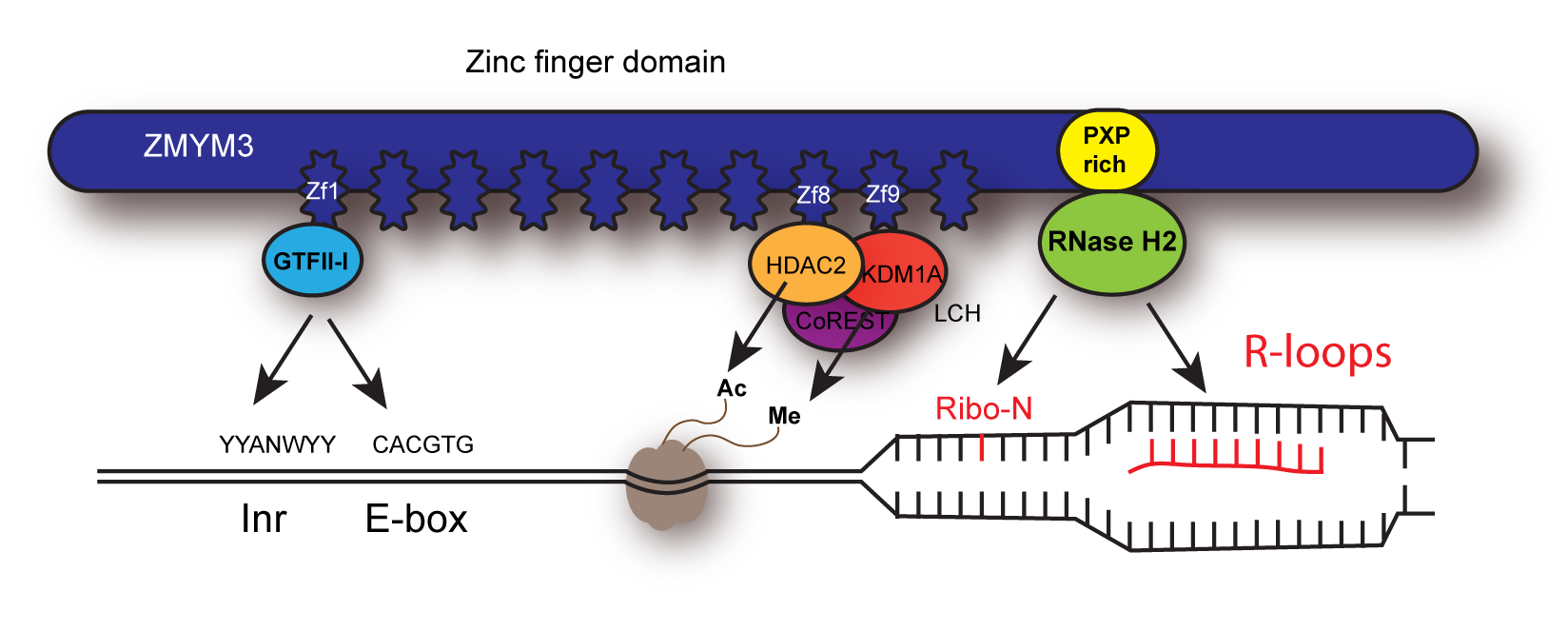

Supplement: S6 Fig — A schematic linear representation of ZMYM3 as a modular scaffold for an array of proteins involved in chromatin modification and recognition. The zinc finger 1 domain is involved in the interaction with General Transcription Factor IIi (GTFII-I) which can recognize DNA in a sequence specific manner (though binding to promoters containing Inr initiator and E-box motifs) whereas the KDM1A/CoREST/HDAC2 LCH complex associates with the central region of the protein through zinc fingers 8 and 9. The C-terminal portion of the protein can recruit RNase H2 to chromatin and DNA though the PXP proline rich domain. This provides a mechanism to coordinate histone tail modification by the LSD1/KDM1A demethylase, histone deacetylation by HDAC2 and transcriptional silencing by CoREST with RNA/DNA hybrid recognition and removal via the RNase activity. (TIF) [file pone.0213553.s008.tif]

Related to Figure 1D:

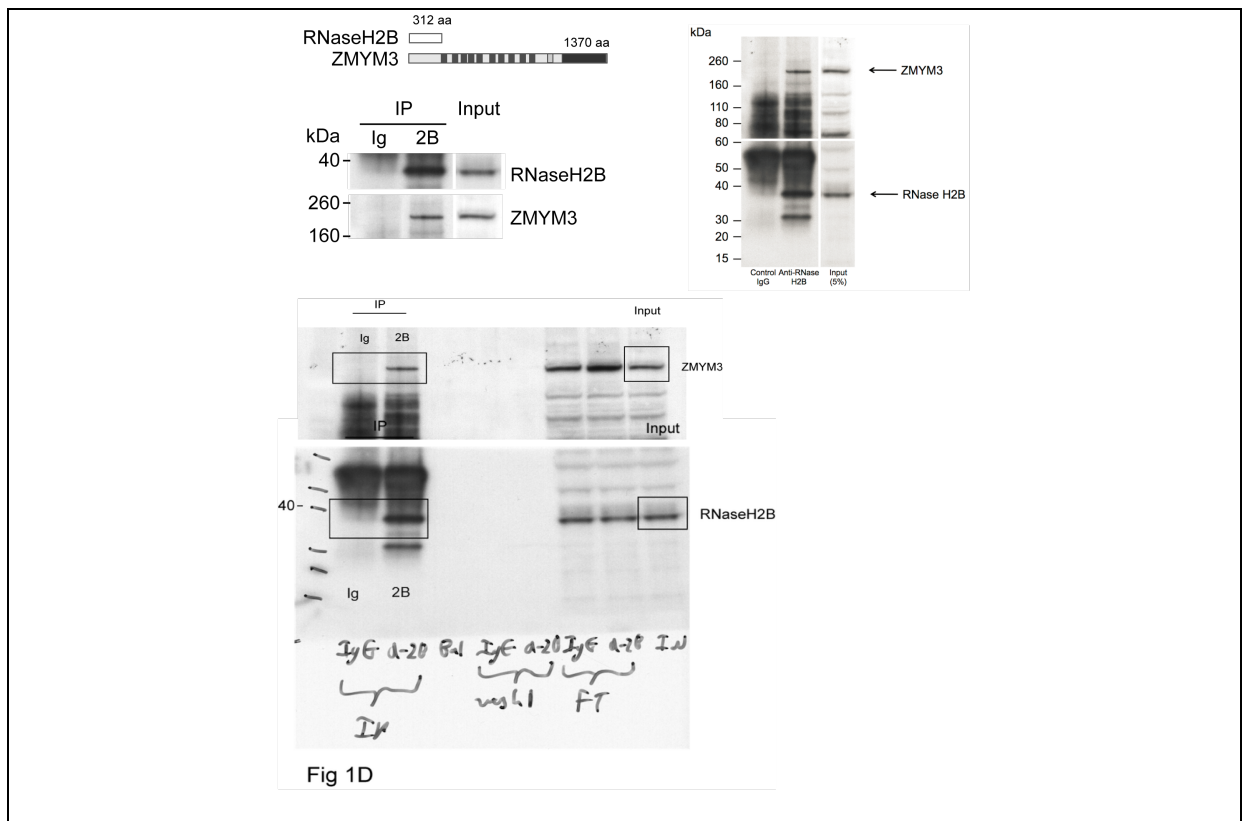

Related to Figure 2B and 2 C:

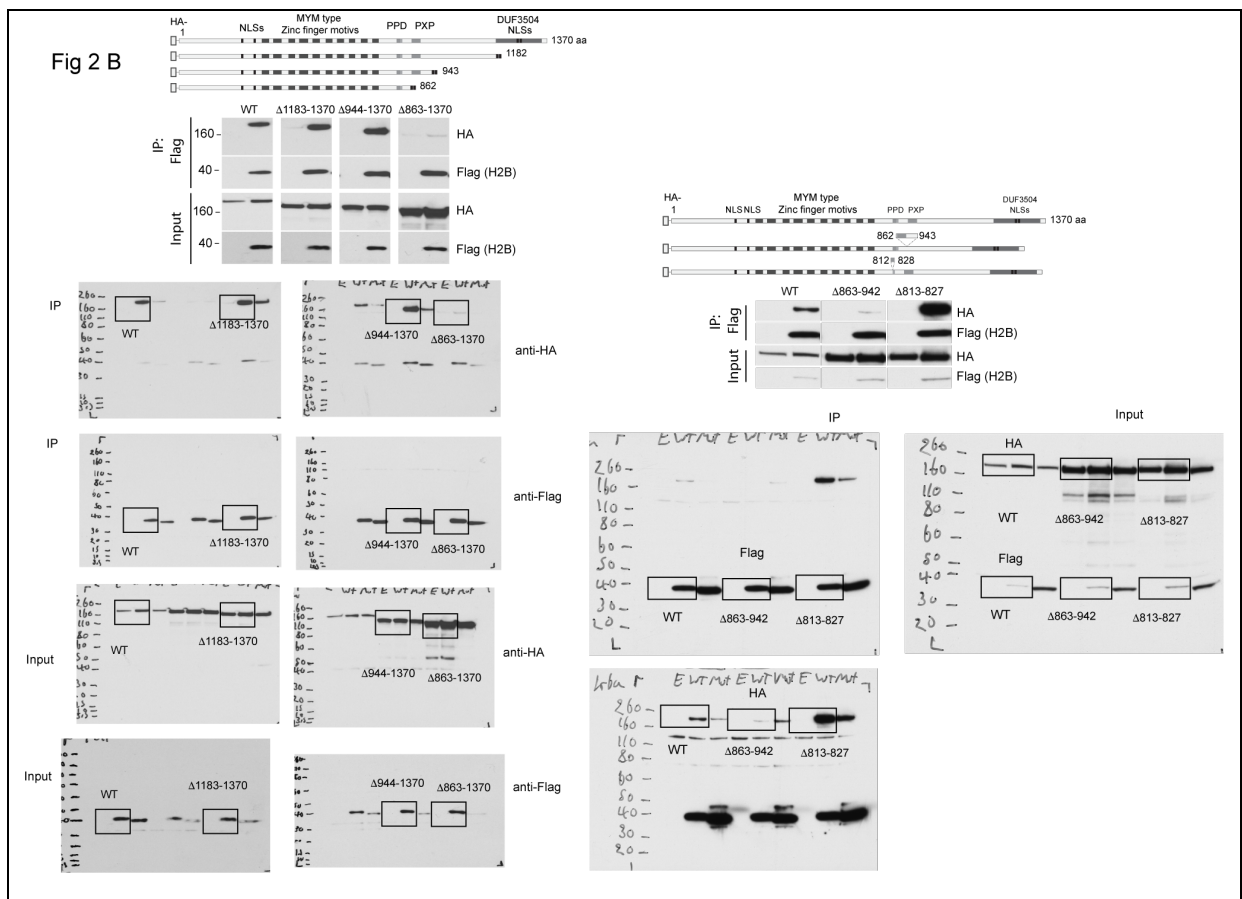

Related to Figure 3 A and 3 B

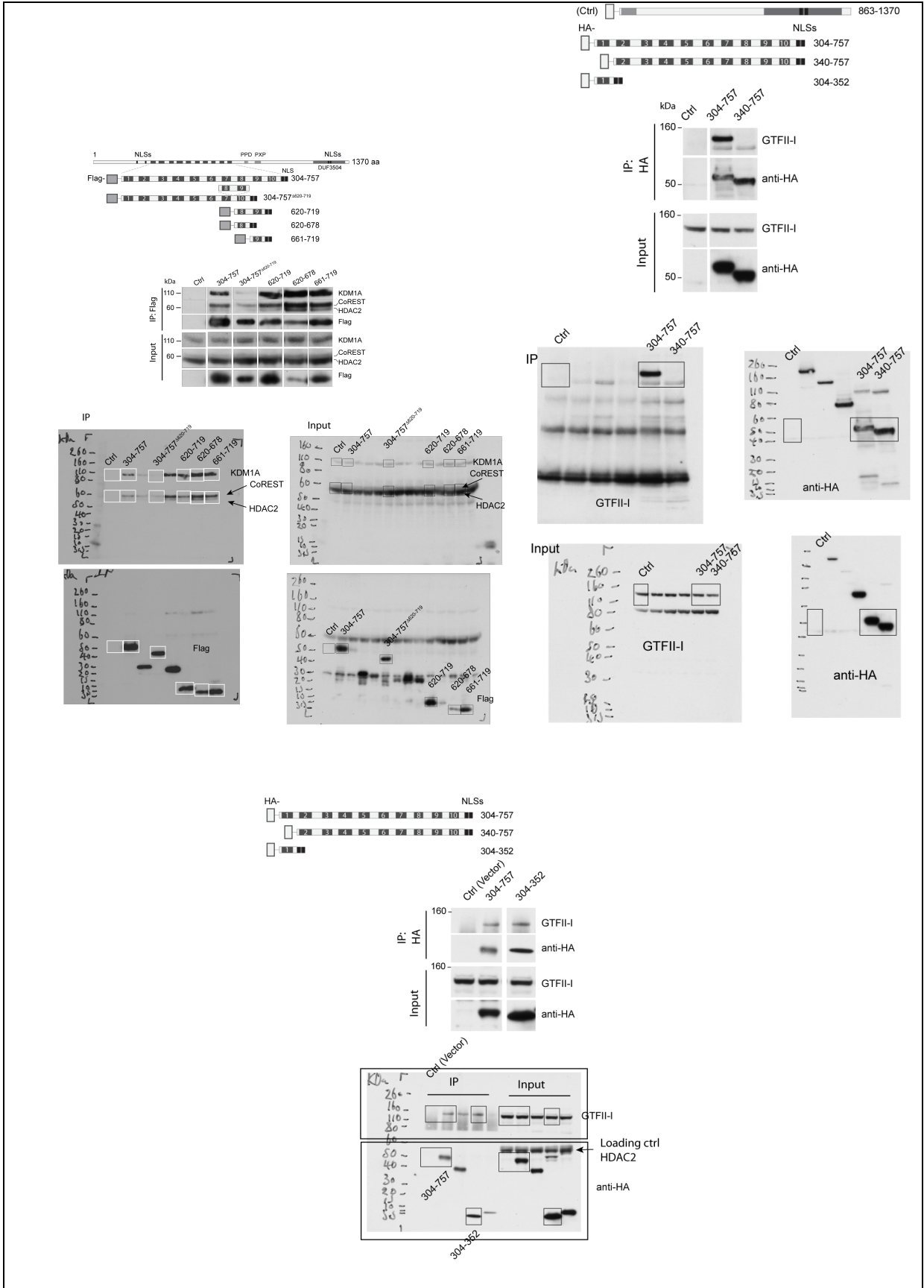

Related to Figure 4B, 4C and 4D:

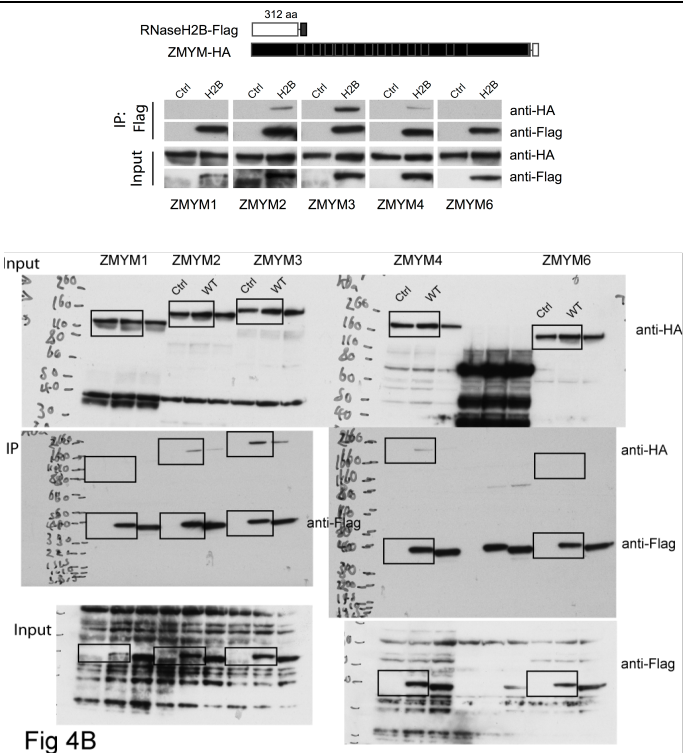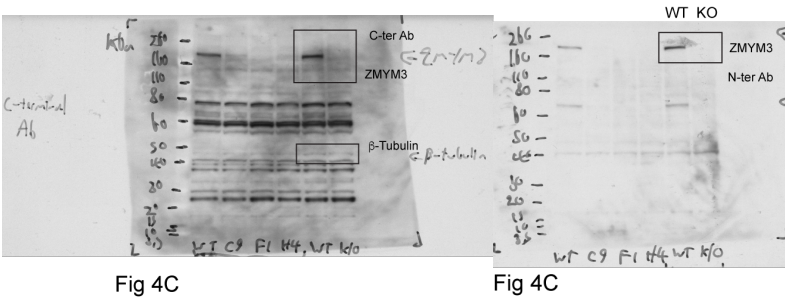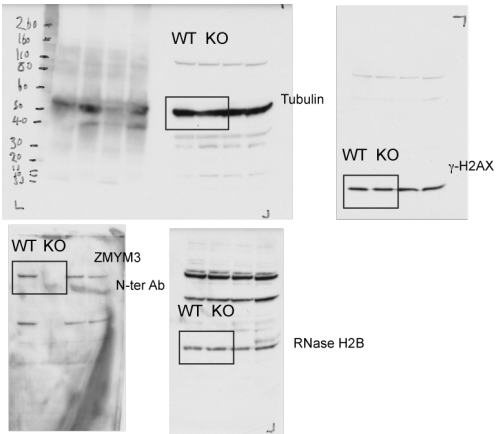

Supplement: S7 Fig — The source images used for each figure are shown and the regions selected to composed the corresponding figures are indicated with the same labelling as used in the main figures. (PDF) [file pone.0213553.s009.pdf]
